# CD4^+^CD25^+^ Regulatory T Cell Depletion Modulates Anxiety and Depression-Like Behaviors in Mice

**DOI:** 10.1371/journal.pone.0042054

**Published:** 2012-07-31

**Authors:** Soo-Jeong Kim, Hyojung Lee, Gihyun Lee, Sei-Joong Oh, Min-Kyu Shin, Insop Shim, Hyunsu Bae

**Affiliations:** 1 Department of Physiology, College of Oriental Medicine, Kyung-Hee University, Seoul, Republic of Korea; 2 Acupuncture and Meridian Science Research Center, College of Oriental Medicine, Kyung-Hee University, Seoul, Republic of Korea; Emory University, United States of America

## Abstract

Stress has been shown to suppress immune function and increase susceptibility to inflammatory disease and psychiatric disease. CD4^+^CD25^+^ regulatory T (Treg) cells are prominent in immune regulation. This study was conducted to determine if anti-CD25 antibody (Ab) mediated depletion of Treg cells in mice susceptibility to stress-induced development of depression-like behaviors, as well as immunological and neurochemical activity. To accomplish this, an elevated plus-maze test (EPM), tail suspension test (TST), and forced swim test (FST) were used to examine depression-like behaviors upon chronic immobilization stress. Immune imbalance status was observed based on analysis of serum cytokines using a mouse cytometric bead array in conjunction with flow cytometry and changes in the levels of serotonin (5-HT) and dopamine (DA) in the brain were measured by high performance liquid chromatography (HPLC). The time spent in the open arms of the EPM decreased significantly and the immobility time in the FST increased significantly in the anti-CD25 Ab-treated group when compared with the non stressed wild-type group. In addition, interlukin-6 (IL-6), tumor necrosis factor-á (TNF-á), interlukin-2 (IL-2), interferon-gamma (IFN-γ), interlukin-4 (IL-4) and interlukin-17A (IL-17A) concentrations were significantly upregulated in the stressed anti-CD25 Ab-treated group when compared with the non stressed wild-type group. Furthermore, the non stressed anti-CD25 Ab-treated group displayed decreased 5-HT levels within the hippocampus when compared with the non stressed wild-type group. These results suggest that CD4^+^CD25^+^ Treg cell depletion modulated alterations in depressive behavior, cytokine and monoaminergic activity. Therefore, controlling CD4^+^CD25^+^ Treg cell function during stress may be a potent therapeutic strategy for the treatment of depression-like symptoms.

## Introduction

Stressful experiences, particularly chronic and unintended stressors, are significant risk factors that play a pervasive role in the etiology of myriad of diseases that they produce and exacerbate. Chronic stress is associated with a neurobehavioral syndrome that is suggestive of depression and multiple processes ranging from psychic-related disorders to activation of the inflammatory immune system [1], [2], [3]. Several studies have consistently indicated that psychological, behavioral and neurobiological profiles of depression are linked to the effects of inflammatory cytokines. [4], [5], [6]. However, the pathophysiologic mechanism of depression is still largely unknown in regards to the interactions between the nervous and immune system.

CD4^+^CD25^+^ Regulatory T (Treg) cells are a subset of thymus-derived CD4^+^ T cell populations that play a crucial role in maintaining immune homeostasis and tolerance by inhibiting the proliferation and the production of cytokines via their constitutive expression of the IL-2 receptor α-chain (CD25) and the transcription factor Foxp3 [7], [8], [9]. There is a great deal of convincing data demonstrating that CD4^+^CD25^+^ Treg cells suppress the development of chronic inflammatory diseases, such as lupus, rheumatoid arthritis [10], [11] and multiple sclerosis [12] through different mechanisms concurrently and sequentially [13]. One mechanism through which stressors might affect numerous processes aligned with neurobehavioral syndrome reminiscent of depression is by activation of the inflammatory immune system [1], [3]. Chronic inflammation may also be associated with both stress and impaired humoral immunity through the improper functioning of helper T cells [14], [15]. However, the role of CD4^+^CD25^+^ Treg cells in major depression, which also exhibits immune imbalance, has not yet been explored.

There has been increasing interest in understanding the relationship between activation of immune response, such as the release of immune cytokines, and the development of neuropsychiatric disorders, including major depression [16], [17]. Indeed, patients with major depression have repeatedly been found to have deregulation of the immune system, as indicated by unbalanced peripheral blood inflammatory biomarkers [6] and increased expression of acute phase proteins [18], [19], chemokines and adhesion molecules [20]. These changes have been considered in terms of the imbalance between pro- and anti-inflammatory cytokines, referred to as Th1/Th2 cytokines. In addition, therapeutic administration of the cytokine IL-2 and interferon-á has been shown to lead to depression in patients [21], [22]. However, very little is known about the immunological role that CD4^+^CD25^+^ Treg cells play in stress models of depression.

This study was conducted to assess the possibility that CD4^+^CD25^+^ Treg cells might contribute to the behavioral and biological alterations provoked by chronic immobilization stress (CIS), which has been used to model neuropsychiatric pathology in mice. The influence of CD4^+^CD25^+^ Treg cells deficiency on CIS-induced development of depression-like behaviors in mice was examined. In addition, to identify whether these behavioral effects were accompanied by changes in the circulating cytokine levels and central monoamine activity within several stressor-sensitive brain regions that have been implicated in depression and anxiety, the serum levels of the pro-inflammatory cytokines (IL-6 and TNF-á), Th1 cytokine (IL-2 and IFN-γ), Th2 inflammatory cytokine (IL-4) and Th17 cytokine (IL-17A) and monoamine (DA and 5-HT) within the prefrontal cortex and hippocampus were measured during the development of behavioral responses-related depression. The use of this animal model may further clarify the role of helper T cells and provide insight into the effects of CD4^+^CD25^+^ Treg cells on the susceptibility to the induction of depressive disorders.

## Methods

### Ethics Statement

All experiments were approved by the University of Kyung Hee Animal Care and Use Committee (KHUASP (SE) - 11 - 027).

### Animals

C57BL/6 mice (6 to 8 wk of age, weighing 20 to 25 g) were obtained from Charles River Laboratories international, Inc. (South Korea). Mice were randomly divided into four groups: non-stressed wild-type (WT) mice (naïve + control group, n = 15), stressed WT mice (stress + control group, n = 14), non-stressed CD4^+^CD25^+^ regulatory T (Treg) cells depleted mice (naïve + anti-CD25 antibody -treated group, n = 16), stressed CD4^+^CD25^+^ Treg cells depleted mice (stress + anti-CD25 antibody-treated group, n = 15). Three separate cohorts of mice (n = 5–6/group, 4 groups in one cohort) were assessed in an elevated plus-maze test, tail suspension test, and forced swim test. Among them, the flow cytometric analysis and the serum cytokine concentrations in two cohorts of mice were measured, while the levels of brain monoamines were examined in the remaining cohort after finishing the behavior tests. The mice were group-housed under pathogen-free conditions at a controlled temperature (22–24°C) and 12 h light/dark cycle. The lights remained on from 8∶00 to 20∶00 and food and water were available *ad libitum*.

### Depletion of CD4^+^CD25^+^ Regulatory T Cells in vivo

**Figure 1:**
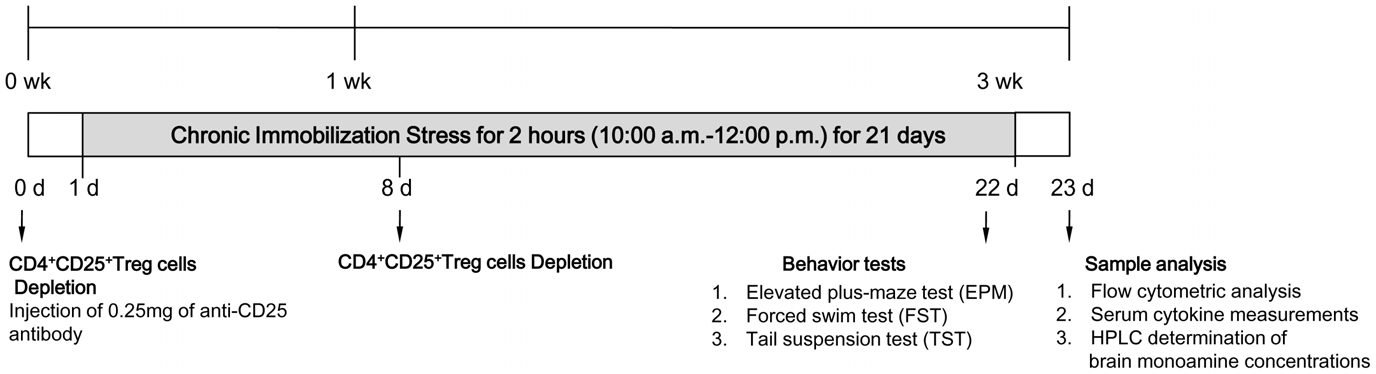
Schedule of experimental procedures. Mice were divided into non-stressed WT mice, stressed WT mice, non-stressed CD4^+^CD25^+^ regulatory T (Treg) cells depleted mice, stressed CD4^+^CD25^+^ Treg cells depleted mice; from day 1 to 22, the stressed WT mice and stressed CD4^+^CD25^+^ Treg cells depleted mice received daily immobilization stress for 4 h and then were moved back to the home cage (three mice to a cage). An anti-CD25 antibody (Ab) was injected intraperitoneally once 24 h before CIS and then on the 8^th^ day after the first CIS. On day 22, EPM, FST and TST were conducted in the afternoon (22 day) to determine depression and anxiety related behaviors after the previous day’s morning CIS.

Anti-mouse CD25 rat IgG1 (anti-CD25; clone PC61) were generated in-house from hybridomas obtained from the American Type Culture Collection (Mamassas, VA, USA). A dose of 0.25 mg of anti-CD25 antibody (Ab) was injected intraperitoneally once 24 h before CIS and then on the 8^th^ day after the first immobilization stress. The efficacy of CD4^+^CD25^+^ Treg cells depletion was confirmed by flow cytometry analysis using PE-anti-mouse CD25 and fluorescent isothiocyanate- anti-mouse CD4 (Fig. 1).

### Chronic Immobilization Stress Procedure

Mice were randomly assigned to a chronic immobilization stressed group or a non-stressed control group. Stress was produced by forcing the animals into an immobilizer device (a disposable rodent restraint cone, Cat. no 529586, Harvard Instruments, Holliston, MA, U.S.A.) for 2 hours (10∶00 a.m.– 12∶00 p.m.) every day for 21 consecutive days. The mouse was eased into the restraint head first, after which the transparent perforated plastic tube was closed, while leaving a nose-hole for breathing. The mice fit tightly into the apparatus to prevent them from moving or turning around. Mice were allowed to habituate to the testing room for at least 1-h before performing the behavior tests. The chronic immobilization stressor used in our study produced typical depressive like behaviors, such as increased duration of immobility and did not decrease locomotive activity on day 21, at which time sickness was not evident [23].

### Behavioral Assessments

Depression and anxiety related behavior testing was conducted on the afternoon (22 day) after the previous day’s morning CIS. Mice were allowed to acclimate to the relevant testing room for at least 1 hour before performing the sequence of behavior tests in order to minimize the acute effects of the experimental stressors, and all apparatuses were thoroughly cleaned between trials/mice with a 70% ethanol solution to minimize the effects of lingering olfactory cues. Twenty-four hours after the last behavior test, all mice were rapidly decapitated and blood, spleen and brain of mice from each of the treatment groups were collected. Blood serum and brain specimens were then stored at −80°C for serum cytokine measurements and HPLC monoamine determination.

### Elevated Plus-maze Test

As anxiety-related variables, the time spent and numbers of entries into the open and closed arms at the end (during three weeks) of the stress procedure were measured. The testing procedure used in this study was based on the method described by Lister, with some modifications [24]. The EPM consisted of a black wooden platform with two open arms (30×5 cm) and two closed arms (30×5×15 cm) arranged in the shape of a plus sign. The arms extended from a central platform (5×5 cm). The maze was elevated 40 cm above the floor. The mice were individually placed in a central platform facing a closed arm and allowed to explore the maze freely for 5 min. The frequency of entries into the open arms and closed arms of the maze and time spent in the respective arms was recorded.

### Forced Swim Test

Mice were individually placed in a glass cylinder (20 cm diameter×25 cm high) containing water (24±1°C) with a depth of 15 cm from the bottom. The water level was deliberately made deeper than in the procedure described by Porsolt *et al*. [25] to prevent the mice from supporting themselves by touching the bottom with their hind limbs or tail. All mice were forced to swim for 6 min, and the duration of immobility was observed and measured during the final 4 min of the test. The immobility time was regarded as the time the mouse spent floating in the water without struggling and making only those movements necessary to keep its head above water.

### Tail Suspension Test

The total duration of immobility induced by tail suspension was based on a method described by Steru *et al*. [26] and measured using computerized TST equipment, BSTST2CA, with the BSTST2LOG software (BIOSEB, Boulogne, France). Briefly, mice were isolated in each chamber and suspended upside down by taping their tails to a flat metal bar with adhesive tape. The mice were positioned so that they could not reach the top or sides of the chamber. The mice were left in this position for six minutes.

### Flow Cytometric Analysis

After the behavioral tests, single-cell suspensions of spleenocytes that were incubated with fluorescently tagged Abs directed against a panel of cell surface markers were stained with CD4, CD25 and Foxp3 (eBiosciences, San Diego, CA, USA). The staining was conducted in accordance with the manufacturer's instructions. All FACS data were acquired using a FACSCalibur flow cytometer (BD Biosciences, San Jose, CA, USA) and analyzed using CellQuest Pro (BD Biosciences, San Jose, CA, USA).

### Serum Cytokine Measurements

Blood samples were collected by retro-orbital puncture and allowed to clot for 1–2 hours at 37°C. They were then centrifuged at 1000 *g* for 20 min at 4°C to obtain the serum, which was stored at −70°C until analyzed in the CBA assay. The serum prepared from each blood sample was subjected to duplicate measurements for cytokine analysis using the mouse cytometric bead array (CBA) (Cat. No 560485BD, Biosciences, San Jose, CA, USA) in accordance with manufacturer’s instructions.

### HPLC Determination of Brain Monoamine Concentrations

After the behavioral tests, the prefrontal cortex and hippocampus were dissected from the mice and homogenized in 200 µl of mobile phase using a Bio-vortexer (Bartlesville, OK, USA). Dopamine (DA), dihydroxyphenylacetic acid (DOPAC), homovanillic acid (HVA), serotonine (5-HT), and 5-hydroxyindole acetic acid (5-HIAA) were purchased from Sigma Aldrich (St. Louis, USA). All other chemicals used in this study were of analytical or HPLC grade. The mobile phase was composed of a phosphate buffer with a final pH of 3.2 and contained 150 mM sodium phsphatemonobasic monohydrate, 1.85 mM octanesulfonic acid, 0.2 mM EDTA, 0.01% (v/v) triethylamine, 4% (v/v) methanol and 6% (v/v) acetonitrile. The homogenate was centrifuged (15 min, 1000×g, 4°C; Micro 17R, Hanil Co. Korea) and the supernatants were filtered using 0.45 µm PVDF (target syringe filter, National Scientific, USA). Twenty microliters of the supernatants were then injected into the HPLC-ECD system, which employed a reversed phase HPLC column (4 µm Nova-pack C18; 3.9×150 mm column; Waters Co., MA, USA) for separation of the monoamines and metabolites. HPLC was conducted at a flow rate of 0.8 ml/min (1525 binary pump; Waters Co) and the monoamine and metabolite content was determined using an electrochemical detector (ESA, MA, USA; Coulochem II Model 5300A; model with a guard cell 5020; 300 mV, micro dialysis cell model 5014B; E2+320 mV, E1–100 mV r; 20 nA).

### Protein Assay

To normalize samples for HPLC, the protein content of the samples was determined by the method described by Bradford [27] using Coomassie brilliant blue reagent (Cat. no 500-0203, Bio-Rad Laboratories, Inc. Hercules, CA, USA). Bovine serum albumin (1 mg/ml) was used as a standard.

### Statistical Analysis

Data were calculated and analyzed using 2 (CD4^+^CD25^+^ Treg cells depletion; wild type vs. CD4^+^CD25^+^ Treg cells depletion treatment) ×2 (CIS; non-stressed vs. stressed condition) analysis of variance (ANOVA) and one way ANOVA followed by a *post hoc* Newman-Keuls multiple comparison test when necessary (GraphPad Software, San Diego, CA, USA). Pearson’s correlation coefficients (2-tailed) were used to calculate the relationship between stress condition and changes in cytokine and monoamine concentrations. All data shown represent the means ± S.E. values. A p<0.05 was considered to be significant.

## Results

### CIS Induced Decrease CD4^+^CD25^+^ Treg Population in Stressed WT Mice

**Figure 2:**
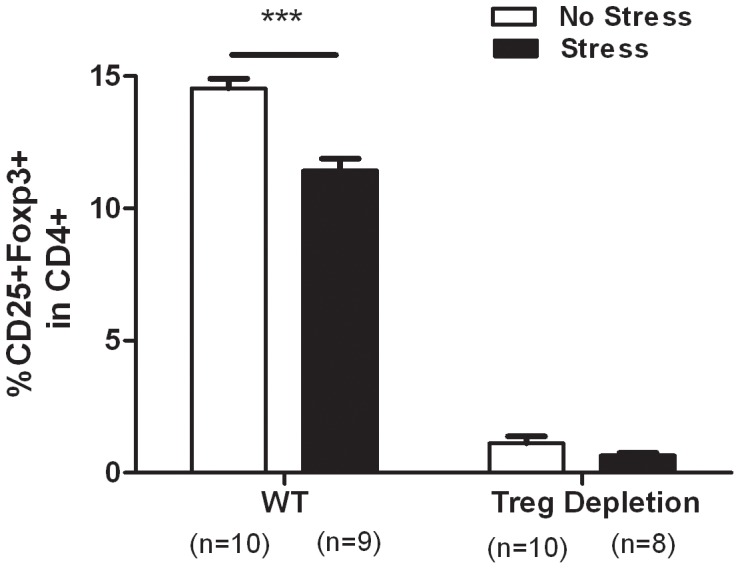
Anti-CD25 Ab-induced depletion of CD4^+^CD25^+^ Treg cells in mice (n = 8–10). Representative dot plots are shown for CD4^+^CD25^+^FOXP3^+^ cells. The efficacy of CD4^+^CD25^+^ Treg cell depletion was confirmed by flow cytometry analysis using PE-anti-mouse CD25 and fluorescent isothiocyanate- anti-mouse CD4 (A). Summaries of the percentage of positive cells are shown in (B) for CD4^+^CD25^+^FOXP3^+^ cells and are presented as the mean ± SE. *** *p*<0.001 *vs.* the wild-type mice.

As shown in Figure 2, stress may decrease the Treg population. Two-way ANOVA analysis revealed a significant interaction between CD4^+^CD25^+^ Treg cell depletion and CIS (*F*_1, 33_ = 15.86, *p* = 0.0004), a significant effect of the CD4^+^CD25^+^ Treg cell depletion (*F*_1, 33_ = 29.23, *p*<0.0001) and effect of the CIS (*F*_1, 33_ = 13.37, *p*<0.0001). Separate one-way ANOVA showed that the CD4^+^CD25^+^ Treg cell population differed significantly among the groups (*F*_3, 33_ = 468.9, *p*<0.0001). The *post hoc* comparisons indicated that the stressed control group had a significantly decreased CD4^+^CD25^+^ Treg cell population when compared with the non-stressed control group (*p*<0.001 ).

### CD4^+^CD25^+^ Treg Cells Depleted Mice Showed Elevated Anxiety-related Behavior

**Figure 3:**
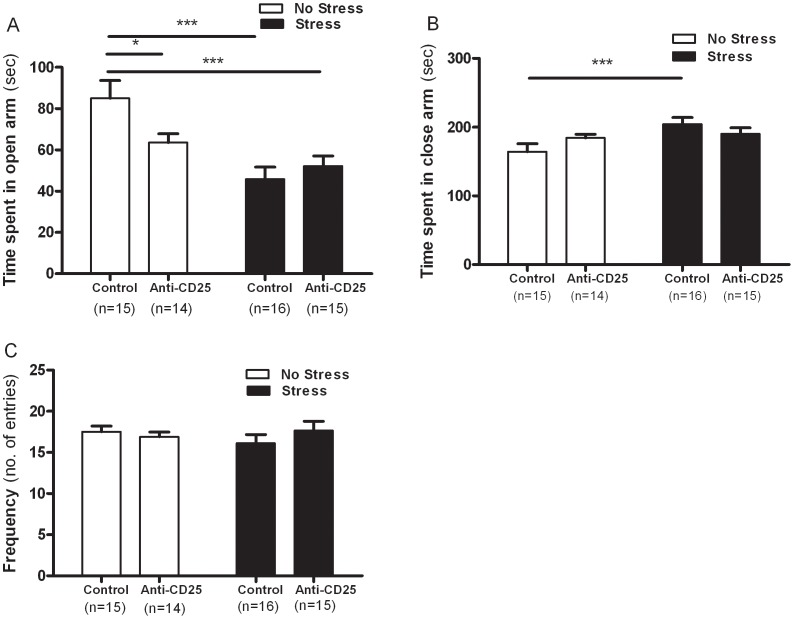
Effects of CD4^+^CD25^+^ Treg cells deficiency upon chronic immobilization stress in the elevated plus maze (n = 14–16). After 21 days of stress, mice were individually placed in a central platform facing a closed arm and allowed to freely explore the maize for 5 min. Time spent in the open arms (A) and closed arms (B) of the maze and the frequency of entry into both arms (C) in the elevated plus maze. The time spent in the respective arms was recorded. Data were analyzed using separate one-way ANOVA followed by a *post hoc* Newman-Keuls test. ***p<0.001, *p<0.05 *vs.* the non-stressed control group. Vertical bars indicate the S.E.

As shown in Figure 3A, two-way ANOVA of the EPM data revealed a significant interaction between CD4^+^CD25^+^ Treg cell depletion and CIS (*F*_1, 56_ = 5.26, *p* = 0.0256) and a significant effect of CIS (*F*_1, 56_ = 17.43, *p* = 0.0001), but there was no significant effect of CD4^+^CD25^+^ Treg cell depletion (*F*_1, 56_ = 17.43, *p* = 0.2173) on time spent in the open arms. These findings indicated that repeatedly stressed mice may display anxiety-related behavior during open space-induced exploration. Separate one-way ANOVA revealed that the time spent in the open arms differed significantly among groups (*F*_3, 56_ = 7.95, *p*<0.001). The *post hoc* comparisons indicated that the stressed control group spent significantly less time in the open arms than the non-stressed control group (*p*<0.001). Similarly, the non-stressed anti-CD25 Ab-treated group (*p*<0.05) and the stressed anti-CD25 Ab-treated group (*p*<0.001) spent significantly less time in the open arms than the non-stressed control group, indicating increased anxiety-related behavior. Concomitant with the data for the open arms, no significant interaction between CD4^+^CD25^+^ Treg cell depletion and CIS (*F*_1, 56_ = 3.35, *p* = 0.0065) was observed in the two-way ANOVA, but the CIS led to significantly elevated time spent in the closed arms of EPM (*F*_1, 56_ = 6.45, *p = *0.0139) without the influence of CD4^+^CD25^+^ Treg cell depletion (*F*_1, 56_ = 0.097, *p* = 0.757) (Fig. 3B). Separate one-way ANOVA revealed that the time spent in the closed arms differed significantly among groups (*F*_3, 56_ = 3.27, *p*<0.05). *Post hoc* comparisons indicated that the stressed control group spent significantly more time in the closed arms than the non-stressed control group (*p*<0.001). Although the anti-CD25 Ab-treated groups showed no significant change in the duration of time spent in the closed arms upon CIS, the anti-CD25 Ab-treated groups tended to spend more time in the closed arms of the EPM. Moreover, the frequency of entries into both arms was unaffected by the interaction between CD4^+^CD25^+^ Treg cell depletion and CIS (*F*_1, 56_ = 1.53, *p* = 0.2243), CIS (*F*_1, 56_ = 0.127, *p* = 0.7231) and CD4^+^CD25^+^ Treg cell depletion (*F*_1, 56_ = 0.301, *p* = 0.5868), indicating that there were no significant differences in the locomotion independent anxiety levels of the mice (Fig. 3C).

### CD4^+^CD25^+^ Treg Cells Depleted Mice Showed Increased Despair Behavior

**Figure 4:**
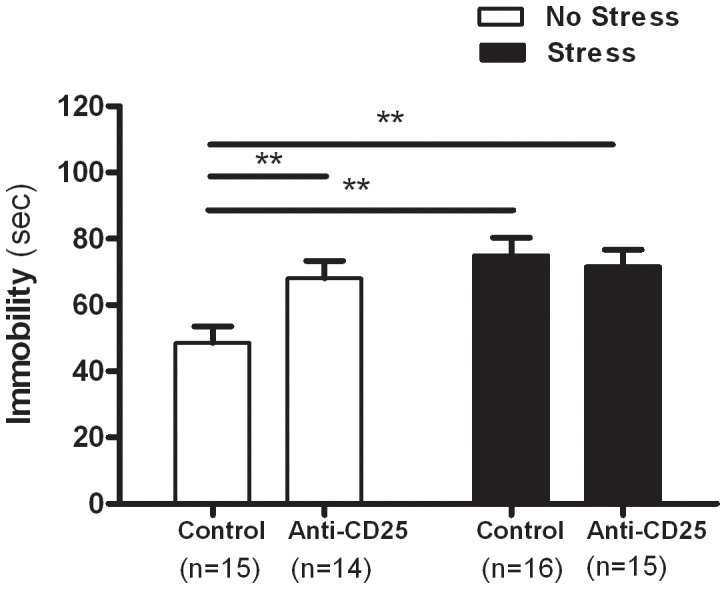
Effects of CD4^+^CD25^+^ Treg cells deficiency on the durations of immobility in the forced swim test (n = 14–16). After 21 days of stress, mice were individually placed in a glass cylinder (20 cm diameter ×25 cm high) containing water. During the 6 min test, the duration of immobility was observed and measured. The immobility time was regarded as the time spent by the mouse floating in the water without struggling. Data were analyzed using separate one-way ANOVA followed by a *post hoc* Newman-Keuls test. ** *p*<0.01 *vs.* the non-stressed control group. Vertical bars indicate the S.E.

A two-way ANOVA of the FST data revealed that the durations of immobility differed as a function of the significant interaction between CD4^+^CD25^+^ Treg cell depletion and the CIS (*F*_1, 56_ = 4.85, *p = *0.0319) and a significant effect of the CIS (*F*_1, 56_ = 8.45, *p* = 0.0052) was observed, as the stressed mice exhibited a markedly depressed phenotype characterized by increased durations of immobility during the FST when compared with non-stressed mice. However, no significant effect of CD4^+^CD25^+^ Treg cell depletion was observed (*F*_1, 56_ = 2.39, *p* = 0.1278) (Fig. 4). Separate one-way ANOVA revealed that the durations of immobility in the FST differed significantly among groups (*F*_3, 56_ = 6.77, *p*<0.01). *Post hoc* comparisons indicated that the stressed control group showed significantly increased durations of immobility in FST when compared with the non-stressed control group (*p*<0.01). Similarly, the stressed anti-CD25 Ab-treated group and the non-stressed anti-CD25 Ab-treated group showed significantly higher durations of immobility in FST when compared with the non-stressed control group (*p*<0.01 and *p*<0.01).

**Figure 5:**
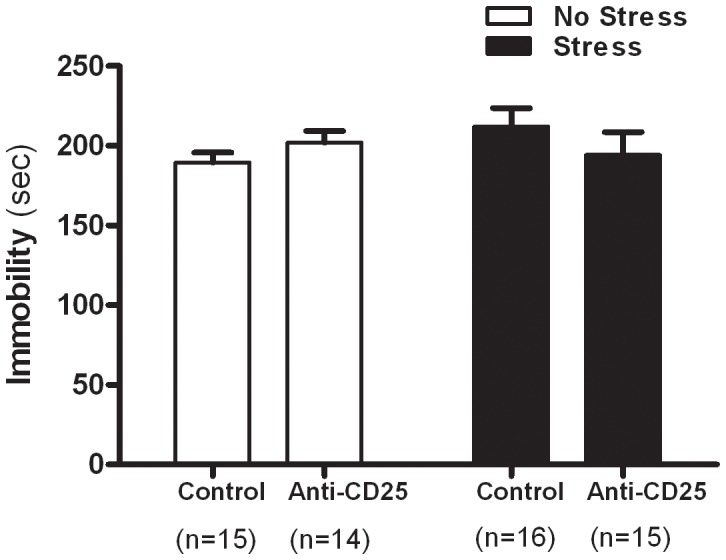
Effects of CD4^+^CD25^+^ Treg cells deficiency on the durations of immobility in the tail suspension test (n = 14–16). After 21 days of stress, mice were isolated in each chamber and suspended upside down by taping their tails to a flat metal bar so that they could not reach the top or the sides of the chamber. The mice were left in this position for 6 min and the total duration of immobility was measured. Data were analyzed using separate one-way ANOVA followed by a *post hoc* Newman-Keuls test. Vertical bars indicate the S.E.

As shown in Figure 5, two-way ANOVA analysis of the TST data showed that the durations of immobility did not differ significantly among groups (CD4^+^CD25^+^ Treg cells depletion×CIS interaction effect, *F*_1, 56_ = 2.17, *p = *0.1462; CIS main effect, *F*_1, 56_ = 0.522, *p = *0.4729; CD4^+^CD25^+^ Treg cells depletion main effect, *F*_1, 56_ = 0.062, *p = *0.8032). Separate one-way ANOVA analysis also showed that the durations of immobility did not differ significantly among groups (*F*_3, 56_ = 0.89, *p*>0.05).

### Effects of the Chronic Immobilization Stress Treatment and CD4^+^CD25^+^ Treg Cells Deficiency on Serum Cytokines

**Figure 6:**
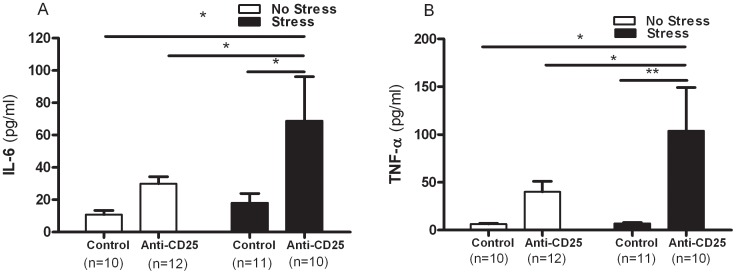
Effects of CD4^+^CD25^+^ Treg cells deficiency on serum concentrations of the pro-inflammatory cytokines IL-6 (A), and TNF-á (B) (n = 10–12). After the behavior tests, blood samples were collected from the mice by retro-orbital puncture. The serum prepared from each blood sample was then subjected to duplicate measurements for cytokine analysis using the mouse cytometric bead array (CBA). Data were analyzed using separate one-way ANOVA followed by a *post hoc* Newman-Keuls test. ** *p*<0.01, * *p*<0.05 *vs*. the stressed anti-CD25 Ab-treated group. Vertical bars indicate the S.E.

As shown in Figure 6A and 6B, a significant effect of CD4^+^CD25^+^ Treg cell depletion on serum concentrations of the monocytic pro-inflammatory cytokines, IL-6 (*F*_1, 39_ = 6.86, *p* = 0.0125) and TNF-α (*F*_1, 39_ = 8.97, *p* = 0.0048) were observed in the two-way ANOVA, but there were no significant interaction between CD4^+^CD25^+^ Treg cell depletion and CIS and the effect of CIS on the concentrations of IL-6 (*F*_1, 39_ = 1.40, *p* = 0.2433 and *F*_1, 39_ = 3.01, *p* = 0.0908) and TNF-α (*F*_1, 39_ = 2.10, *p* = 0.1555 and *F*_1, 39_ = 2.09, *p* = 0.0.1471). Furthermore, the magnitude of the increase in IL-6 and TNF-α concentration in response to the stressor differed significantly among the control and anti-CD25 Ab-treated mice. Separate one-way ANOVA revealed that the concentrations of IL-6 and TNF-α differed significantly among groups (*F*_3, 39_ = 3.58, *p*<0.05 and *F*_3, 39_ = 4.26, *p*<0.01). *Post hoc* comparisons indicated that the stressed anti-CD25 Ab-treated group had higher serum IL-6 and TNF-α concentrations when compared with the non-stressed control group (*p*<0.05 and *p*<0.05) and the stressed control group (*p*<0.05 and *p*<0.05). In addition, the stressed anti-CD25 Ab-treated group showed significantly higher IL-6 and TNF-α concentration when compared with the non-stressed anti-CD25 Ab-treated group (*p*<0.05 and *p*<0.05).

**Figure 7:**
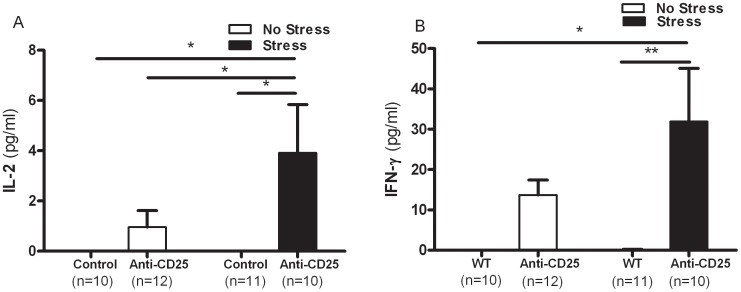
Effects of CD4^+^CD25^+^ Treg cells deficiency on serum concentrations of the Th1 cytokines IL-2 (A) and IFN-γ (B) (n = 10–12). After the behavior tests, blood samples were collected from the mice by retro-orbital puncture. The serum prepared from each blood sample was then subjected to duplicate measurements for cytokine analysis using the mouse cytometric bead array (CBA). Data were analyzed using separate one-way ANOVA followed by a *post hoc* Newman-Keuls test. ** *p*<0.01, * *p*<0.05 *vs*. the stressed anti-CD25 Ab-treated group. Vertical bars indicate the S.E.

Similarly, two-way ANOVA revealed that the serum concentrations of Th1 cytokine, IL-2 and interferon-gamma (IFN-γ) were significantly affected by the depletion of CD4^+^CD25^+^ Treg cells (*F*_1, 39_ = 6.53, *p* = 0.0146 and *F*_1, 39_ = 12.47, *p* = 0.0011), but there were no significant interaction between CD4^+^CD25^+^ Treg cells depletion and CIS (IL-2; *F*_1, 39_ = 2.41, *p* = 0.1290 and IFN-γ; *F*_1, 39_ = 1.98, *p* = 0.1677) and a significant effect of CIS was observed (IL-2; *F*_1, 39_ = 2.41, *p* = 0.1290 and IFN-γ; *F*_1, 39_ = 2.03, *p* = 0.1626). Separate one-way ANOVA revealed that the concentrations of IL-2 and IFN-γ differed significantly among groups (*F*_3,39_ = 3.63, *p*<0.05, and *F*_3,39_ = 5.33, *p*<0.01), as the stressed anti-CD25 Ab-treated group had higher levels of IL-2 and interferon-gamma (IFN-γ) when compared with the non-stressed control group (*p*<0.05 and *p*<0.05) and the stressed control group (*p*<0.05 and *p*<0.01). The stressed anti-CD25 Ab-treated group also had elevated IL-2 concentrations when compared with the non-stressed anti-CD25 Ab-treated group (*p*<0.05) (Fig. 7A and Fig. 7B).

**Figure 8:**
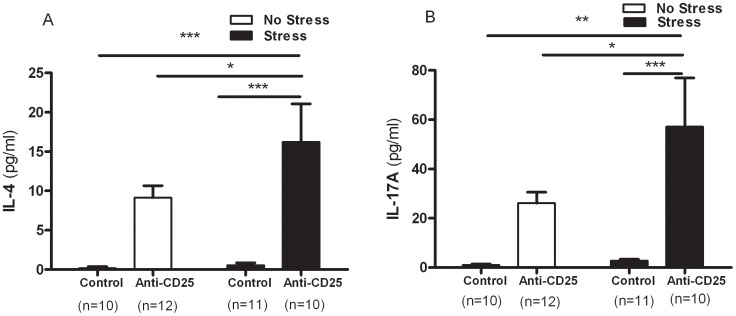
Effects of CD4^+^CD25^+^ Treg cells deficiency on serum concentrations of the Th2 cytokine IL-4 (A) and the Th17 cytokine IL-17A (B) (n = 10–12). After the behavior tests, blood samples were collected from the mice by retro-orbital puncture. The serum prepared from each blood sample was subjected to duplicate measurements for cytokine analysis using the mouse cytometric bead array (CBA). Data were analyzed using separate one-way ANOVA followed by a *post hoc* Newman-Keuls test. *** *p*<0.00, ** *p*<0.01, * *p*<0.05 *vs*. the stressed anti-CD25 Ab-treated group. Vertical bars indicate the S.E.

As shown in Figure 8A, a significant effect of CD4^+^CD25^+^ Treg cell depletion on the serum concentrations of IL-4 was observed in two-way ANOVA analysis, which differed significantly among groups (*F*_1, 39_ = 26.51, *p* = 0.001), but there were no significant interaction between CD4^+^CD25^+^ Treg cell depletion and CIS (*F*_1, 39_ = 1.97, *p* = 0.1689) and a significant main effect of CIS (*F*_1, 39_ = 2.43, *p* = 2.978) was not observed. In addition, IL-17A varied as a function of the significant effect of CD4^+^CD25^+^ Treg cell depletion (*F*_1, 39_ = 26.51, *p* = 0.0001) and CIS (and *F*_1, 39_ = 17.56, *p* = 0.002), but there was no significant interaction between CD4^+^CD25^+^ Treg cell depletion and CIS (*F*_1, 39_ = 2.38, *p* = 0.1311) in the serum concentrations of IL-17A (Fig. 8B). Separate one-way ANOVA showed a significant effect of the differences in concentrations of IL-4 and IL-17A between the control and anti-CD25 Ab-treated group (*F*_3, 39_ = 10.05, *p*<0.001 and *F*_3, 39_ = 7.39, *p*<0.001). *Post hoc* comparisons indicated that the serum concentrations of IL-4 and IL-17A of the stressed anti-CD25 Ab-treated group were elevated relative to the non-stressed control group (*p*<0.001 and *p*<0.01), the stressed control group (*p*<0.001and *p*<0.001) and the non-stressed anti-CD25 Ab-treated group (*p*<0.05 and *p*<0.05).

### CD4^+^CD25^+^ Treg Cells Depleted Mice Displayed Decreased 5-HT Levels within the Hippocampus

**Figure 9:**
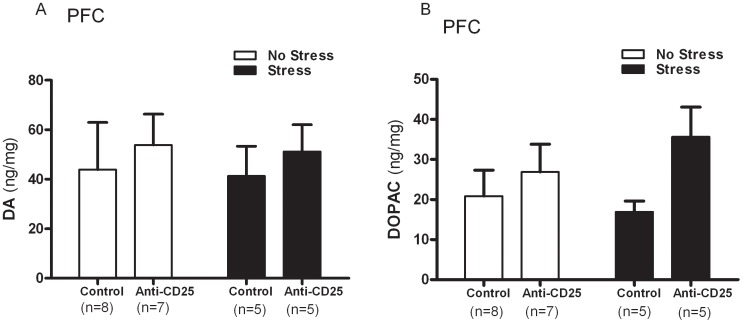
Effects of CD4^+^CD25^+^ Treg cells deficiency on levels of dopamine (DA) (A) and its metabolite, DOPAC (B) within the prefrontal cortex (n = 5–8). After the behavioral tests, the prefrontal cortex and hippocampus were dissected from the mice and homogenized. Each supernatant sample was then injected into a reversed phase HPLC column, after which the amounts of monoamines and metabolites were determined using an electrochemical detector. Data were analyzed by separate one-way ANOVA followed by a *post hoc* Newman-Keuls test. Vertical bars indicate S.E.

**Figure 10:**
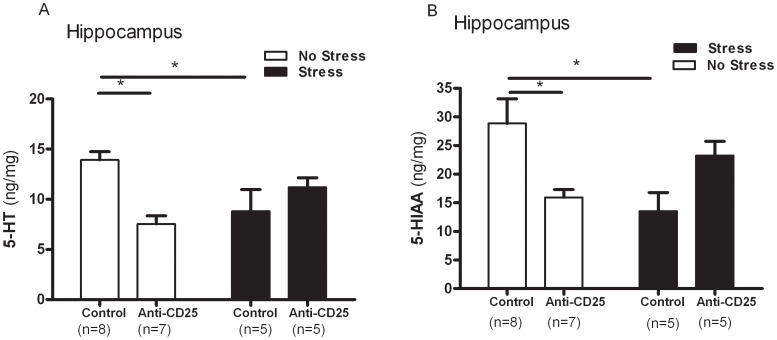
Effects of CD4^+^CD25^+^ Treg cells deficiency on levels of serotonin (5-HT) (A) and its metabolite, 5-HIAA, (B) within the hippocampus (n = 5–8). After the behavioral tests, the prefrontal cortex and hippocampus were dissected from the mice and homogenized. Each supernatant sample was injected into a reversed phase HPLC column. The amounts of monoamines and metabolites were then determined using an electrochemical detector. Data were analyzed using separate one-way ANOVA followed by a *post hoc* Newman-Keuls test. * *p*<0.05 *vs.* the non-stressed control group. Vertical bars indicate the S.E.

Two-way ANOVA revealed that neither the DA levels (CD4^+^CD25^+^ Treg cells depletion×CIS interaction effect, *F*_1, 21_ = 0.01, *p = *0.9999; CIS main effect, *F*_1, 21_ = 0.04, *p = *0.8537; CD4^+^CD25^+^ Treg cells depletion main effect, *F*_1, 21_ = 0.52, *p = *0.4837) nor those of its metabolite, DOPAC (CD4^+^CD25^+^ Treg cells depletion×CIS interaction effect, *F*_1, 21_ = 1.12, *p = *0.3085; CIS main effect, *F*_1, 21_ = 0.16, *p = *0.6959; CD4^+^CD25^+^ Treg cells depletion main effect, *F*_1, 21_ = 4.18, *p = *0.0602) within the prefrontal cortex (PFN) were significantly affected by CD4^+^CD25^+^ Treg cell depletion or the CIS (Fig. 9A and Fig. 9B). Moreover, the changes in 5-HT and its metabolite, 5-HIAA, within the hippocampus in response to the repeated stress treatment differed as a function of the significant interaction between the CD4^+^CD25^+^ Treg cell depletion and the CIS among the control group and anti-CD25 Ab-treated group (*F*_1, 21_ = 5.02, *p = *0.0359 and *F*_1, 21_ = 4.472, *p = *0.0466), but no significant effect of the CD4^+^CD25^+^ Treg cell depletion or CIS on the 5-HT (*F*_1, 21_ = 0.67, *p = *0.4236 and *F*_1, 21_ = 0.003, *p = *0.9523) and 5-HIAA levels (*F*_1, 21_ = 0.06, *p = *0.8040 and *F*_1, 21_ = 1.72, *p = *0.2043) were observed. Separate one-way ANOVA analysis revealed that the level of 5-HT and its metabolite 5-HIAA within the hippocampus differed significantly between the control group and anti-CD25 Ab-treated group (*F*_3, 21_ = 4.26, *p*<0.05 and *F*_3, 21_ = 4.39, *p*<0.05). *Post hoc* comparisons indicated that the stressed anti-CD25 Ab-treated group and the non-stressed anti-CD25 Ab-treated group showed significantly reduced 5-HT levels within the hippocampus when compared with the non-stressed control group (*p*<0.05). The 5-HIAA levels were greatly reduced in the stressed control group and the non-stressed anti-CD25 Ab-treated group when compared to the non-stressed control group (*p*<0.05) (Fig. 10A and Fig. 10B).

### Correlation between Stress-induced Behavior and Inflammatory Cytokines/monoamines

**Figure 11:**
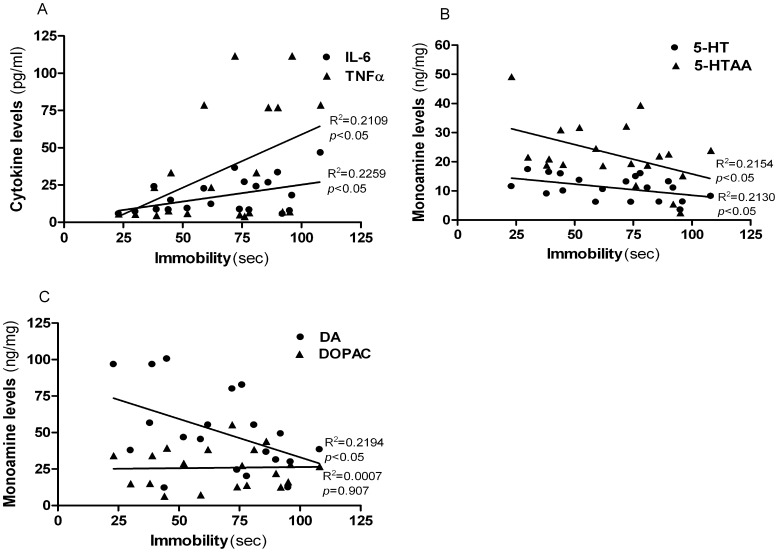
Correlation between the durations of immobility in the forced swim test and levels of cytokines and monoamines (B, C). Correlations in immobility are shown in the scatter-plat with IL-6 and TNF-á (*Closed circles = *IL-6: *closed triangles = *TNF-á) (A), 5-HT and 5-HIAA (*Closed circles = *5-HT : *closed triangles = *5-HIAA) (B) and DA and DOPAC (*Closed circles = *DA : *closed triangles = *DOPAC) (C) plotted separately. Data were analyzed using Pearson’s correlation coefficients (2-tailed).There were significant correlation at the level of 0.05 between the stress condition and changes in cytokines (IL-6, r = 0.48 and TNF-á, r = 0.46) and monoamines (5-HT, r = −0.46; 5-HIAA, r = −0.46; DA, r = −0.47) concentrations.

When all of the animals were included in the correlation analysis, the immobility of FST as CIS-related variables was found to be positively correlated with the levels of IL-6 (r = 0.48, *p* = 0.0342) and TNF-á (r = 0.46, *p* = 0.0417) (Fig. 11A). There was no correlation between the immobility of FST and IL-2 (r = 0.23, *p = *0.3360), IFN-γ (r = 0.35, *p* = 0.1301), IL-4 (r = 0.40, *p* = 0.0842) and IL-17A (r = 0.33, *p* = 0.0842). The immobility of FST was also negatively correlated with 5-HT (r = −0.46, p = 0.0405), 5-HIAA (r = −0.46, p = 0.0393) of hippocampus (Fig. 11B). Furthermore, we found a negative correlation between the immobility of FST and DA of the prefrontal cortex (r = −0.47, *p* = 0.0372) (Fig. 11C).

## Discussion

The results of the present study demonstrates that CD4^+^CD25^+^ Treg cell depleted mice displayed markedly increased anxiety in the open arms of the EPM and immobility in the FST. In addition, the serum concentrations of the pro-inflammatory cytokines (IL-6 and TNF-á), Th1 cytokines (IL-2 and IFN-γ), Th2 inflammatory cytokine (IL-4) and Th17 cytokine (IL-17A) were significantly elevated in CD4^+^CD25^+^ Treg cell depleted mice. Moreover, a significant decrease in 5-HT levels within the hippocampus was observed in the CD4^+^CD25^+^ Treg cell depleted mice. We found that CIS led to a decrease in the CD4^+^CD25^+^ Treg population in the splenocytes of WT mice that received the CIS. This observation is consistent with the finding that the immune imbalance in major depression patients resulted in a decrease in the Treg cell population in peripheral blood lymphocyte [28]. Tregs have been also known to inhibit the extenuating effects of autoreactive T cells on stress-induced anxiety- like behaviors [29]. Many studies have suggested that stress activation of the immune system predominantly produces pro-inflammatory cytokines, which initiates inflammatory immune responses [4], [30], [31]. Previous studies that have examined major depression have shown that elevated IL-2 and IL-6 production could be integrated with an inflammatory response system during a depressive state [32], [33], although some studies have shown conflicting results [34], [35]. Similarly, Lanquillon *et al*. reported that the production of TNF-α by peripheral blood mononuclear cells is significantly higher in depressed patients than in normal control patients [36]. Interestingly, present data also demonstrated that the level of IL-6 and TNF-á was positively correlated with the depressive behavior, supporting the hypothesis that the inflammatory cytokines cause development of depression. Moreover, previous studies have demonstrated that several genes that modulate T cell function are associated with major depression and the response to antidepressants. For example, single nucleotide polymorphisms (SNPs) in the PSMB4 and T-bet (Tbx 21) genes are strongly involved in the susceptibility to major depression [37]. Th1 cell cytokines and IFN-ã production induced by T-bet (Tbx21), which initiates development of the Th1 cell lineage from naive pre-Th cells, was higher in depressed patients and Th2 anti-inflammatory cytokine, IL-4 was lower in normal controls [38], [39], [40], [41]. Our recent study demonstrated that T-bet deficient mice exhibit impaired depression like behavior upon CIS [41]. Consistent with these previous reports, the immune imbalance in major depression patients was not only characterized by changes in cytokine levels, but also by a decrease in the CD4^+^CD25^+^ Treg cell population in peripheral blood lymphocytes. These changes may prove important to understanding the mechanism involved in immune deregulation [39]. The results of the present study suggest that CD4^+^CD25^+^ Treg cell deficiency may contribute to a progressive effect on stress-induced immunological variations. Such findings are consistent with the possibility that stressor-induced increases in circulating pro inflammatory cytokines may occur as a result of the action of Th immune cells, and that CD4^+^CD25^+^ Treg cell deficiency may aggravate the immunological effect of CIS. These findings also suggest that Th cell dysfunction in major depression may not only represent a consequence of the disease, but also plays an important role in its cause.

In the case of the central nervous system (CNS), Treg cells can have a beneficial effect by short circuiting the inflammatory loop of T cells and antigen-presenting cells (APCs) [42]. Moreover, peripheral cytokines may directly trigger vagal and trigeminal afferent fibers and promote cytokines, mainly produced by microglia and astroglia through all CNS cells [43], [44], [45]. Furthermore, cytokines have specific transporter mechanisms at the blood- brain barrier (BBB) [46]. Increased BBB permeability [47] by stress can cause an increase in the infiltration of peripheral immune cells, such as activated T lymphocytes and natural killer cells [48], into brain parenchyma. Interestingly, it has been shown that CD4^+^ T cells were found to be involved in neuroprotection during the pathology of neurodegenerative disorders of the central nervous system (CNS) such as Alzheimer’s (AD) and Parkinson disease (PD) [49], [50], [51]. Neuroinflammation and the progressive loss of nerve cells occur concurrently with activated microglia as part of a local innate immune reaction, production of inflammatory cytokines and limited T cells infiltration [52], . The capacity of CD4^+^ T cells to reduce microglia responses and protect against neurodegeneration suggests the possible involvement of CD4^+^CD25^+^ Treg cells. Therefore, given the potential role of inflammation in depression and the ability of Treg to inhibit inappropriate or excessive immune responses, it is an intriguing possibility that decreased Treg activity may be relevant to the pathogenesis of depression. However, few studies exploring the intracellular mechanisms that regulate communication between stress and T cells have been reported.

In this study, CD4^+^CD25^+^ Treg cell depleted mice were shown to be susceptible to the CIS-induced development of depression-like behaviors, indicating that stress induced depression may be considered a pathogenic immune disorder. Anxiety is a symptom with high prevalence in depression. Therefore, animal models that are used to elucidate mechanisms underlying depression often display altered anxiety-related behavior, which can be assessed in the elevated plus-maze test (EPM) [54]. The time spent in the open arms by CD4^+^CD25^+^ Treg cell depleted mice was significantly reduced when compared to that of non-stressed WT mice, indicating that anxiety-related behaviors were promoted by both CIS and the depletion of CD4^+^CD25^+^ Treg cells. The forced swim test (FST) and the tail suspension test (TST), which are two standard tests used for the assessment of despair behavior, were also conducted. In the FST, mice were forced to swim in a restricted space from which they cannot escape and were induced to assume a characteristic behavior of immobility [25]. The stressed WT mice exhibited markedly increased durations of immobility during the FST when compared with the non-stressed WT mice, while CD4^+^CD25^+^ Treg cell depleted mice were not further influenced by the CIS. In the TST, the animal immediately engaged in several agitation- or escape-like behaviors, followed by increasing bouts of immobility upon suspension by its tail [26]. The stressed WT mice and the CD4^+^CD25^+^ Treg cell depleted mice showed slightly increased durations of immobility in TST when compared to the non-stressed WT mice, but these increases were not significant. These findings indicate that CIS treatment resulted in a marked increase in depression-like behaviors during the FST in WT mice, while Treg depleted mice were no longer responding to CIS with elevated depressive behaviors.

Central monoamines, specifically serotonin (5-HT), norepinephrine (NE) and dopamine (DA), are widely distributed in the brain, and their functional role is well established under stressful conditions. [55]. To ascertain if CD4^+^CD25^+^ Treg cell deficiency contributes to CIS-induced behavioral deficits by moderating the central effects of stress, changes in DA and its metabolites, DOPAC, in the prefrontal cortex, which has a high DA content, were evaluated [56]. In addition, changes in 5-HT and its metabolite, 5-HIAA, present in the hippocampus, which has a high concentration of glucocorticoid (GC) receptors [57] were observed. Noticeable decreases in the hippocampus 5-HT levels were observed in CD4^+^CD25^+^ Treg cell depleted mice. In contrast, DA levels and DOPAC within the prefrontal cortex were not affected by the treatments, indicating a region-specific effect of stress, although an increase in the DA levels in the prefrontal cortex were observed in CD4^+^CD25^+^ Treg cell depleted mice [58], [59], [60]. These changes were significantly positively correlated with CD4^+^CD25^+^ Treg cell deficiency, which indicated that monoamines within the hippocampus may interact with CD4^+^CD25^+^ Treg cells. The results of neurochemical variations indicate that CIS and CD4^+^CD25^+^ Treg deficiency influences 5-HT activity within the hippocampus. It should be noted that reduced monoamine concentrations are relevant to increased stress sensitization and their preferential and higher utilization. A recent study showed that low levels of brain monoamine neurotransmitters, especially 5-HT, could induce depression [61]. A more recent study demonstrated that a decreased in serotonin tissue concentration induced by chronic mild stress was apparent without a subsequent increase in the 5-HT turnover ratio due to the simultaneous decrease in 5- HIAA tissue levels [62]. The reduction in 5-HT levels and its metabolite during CIS is in agreement with the results of studies, which found that a decrease of 5-HT in the brain varied in response to chronic social stress, suggesting that 5-HT may play a role in such processes [63], [64]. We also found a significant correlation between CIS-induced behavior changes and pro-inflammatory cytokines (IL-6 and TNF-á) and monoamines (5-HT, 5-HIAA and DA), indicating that elevated levels of pro-inflammatory cytokines may function in the development of depression.

In conclusion, CD4^+^CD25^+^ Treg cells play a role in the manifestation of depression-like behaviors, immune alterations and changes in the neurochemical status. Therefore, CD4^+^CD25^+^ Treg cells may provide a promising strategy for the development of CD4^+^CD25^+^ Treg cell-based therapies that will alleviate stress induced disorders, including depression.
